# Race and Gender-Based Perceptions of Older Septuagenarian Adults

**DOI:** 10.1089/whr.2022.0063

**Published:** 2022-11-14

**Authors:** Forest Melton, Kelly Palmer, Sade Solola, Luis Luy, Kathryn Herrera-Theut, Leanne Zabala, Shannon M. Knapp, Ryan Yee, Erika Yee, Elizabeth Calhoun, Megan C. Thomas Hebdon, Natalie Pool, Nancy Sweitzer, Khadijah Breathett

**Affiliations:** ^1^ Department of Clinical Translational Sciences, College of Medicine, University of Arizona, Phoenix, Arizona, USA.; ^2^ Department of Promotion Science, College of Public Health, University of Arizona, Tucson, Arizona, USA.; ^3^ Division of Cardiology, Brown University, Providence, Rhode Island, USA.; ^4^ University of Rochester, Rochester, New York, USA.; ^5^ Department of Internal Medicine and Pediatrics, College of Medicine, University of Michigan, Ann Arbor, Michigan, USA.; ^6^ Department of Medicine, University of California, Los Angeles, Los Angeles, California, USA.; ^7^ Division of Cardiovascular Medicine Statistics, College of Medicine, Indiana University, Indianapolis, Indiana, USA.; ^8^ Clinical Research Office, Indiana University, Indianapolis, Indiana, USA.; ^9^ College of Medicine, University of Arizona, Tucson, Arizona, USA.; ^10^ College of Public Health, University of Illinois at Chicago, Chicago, Illinois, USA.; ^11^ School of Nursing, University of Texas at Austin, Austin, Texas, USA.; ^12^ School of Nursing, University of Northern Colorado, Greeley, Colorado, USA.; ^13^ Division of Cardiology, Washington University, St. Louis, Missouri, USA.; ^14^ Division of Cardiovascular Medicine, College of Medicine, Krannert Institute of Cardiology, Indiana University, Indianapolis, Indiana, USA.

**Keywords:** bias, racial disparities, gender disparities, geriatric

## Abstract

**Objectives:**

Older adults face racism, sexism, and ageism. As the U.S. population ages, it is important to understand how the current population views older adults.

**Methods:**

Participants recruited through Amazon's Mechanical Turk provided perceptions of older Black and White models' photographs. Using mixed-effect models, we assessed interactions between race and gender of participants and models.

**Results:**

Among Participants of Color and White participants (*n* = 712, 70% non-Hispanic White, 70% women, mean 37.81 years), Black models were perceived as more attractive, less threatening, and sadder than White models, but differences were greater for White participants (race-by-race interaction: attractive *p* = 0.003, threatening *p* = 0.009, sad *p* = 0.016). Each gender perceived their respective gender as more attractive (gender-by-gender interaction *p* < 0.0001). Male and female participants perceived male models as happier than female models, but differences were greater for male participants (*p* = 0.026). Irrespective of participant age group, women were perceived as more threatening (*p* = 0.012). Other perceptions were not significant.

**Discussion:**

Participants had few biases toward older Black and White models, while gender biases favored men.

## Introduction

Bias is prevalent in society and may disproportionately affect the diverse, aging U.S. population. The number of Americans 65 years and older is projected to double from 52 million in 2018 to 95 million by 2060.^1^ Consequently, the older population is growing more diverse with the non-Hispanic White population being projected to drop from 77% to 55%.^1^ This geriatric population growth, driven by the baby boomer generation, is unprecedented in U.S. history. The negative effects of race and gender bias on Black, Indigenous and People of Color (BIPOC) are further exacerbated by ageism, when considering an older population.^2^ It is well documented that race and gender biases play a significant role in health care disparities.^3–7^ Moreover, BIPOC groups and women experience more bias in the health care system when compared with White individuals and men.^8^ BIPOC individuals may experience worse communication, lower quality of care, and subsequently worsened clinical outcomes as a result of implicit bias, structural racism, and discrimination in health care.^9,10^

Intersectionality is a requisite for understanding perceptions of individuals. Intersectionality was described by Dr. Lisa Bowleg as a theoretical framework, which asserts that multiple social categories (*e.g*., race, ethnicity, gender, age, sexual orientation, socioeconomic status) intersect at the microlevel of individual experience to reflect multiple interlocking systems of privilege and oppression at the macro, social–structural level (*e.g*., racism, sexism, ageism, heterosexism).^11^ Bearing this in mind, it is not difficult to recognize that the resultant sexism, racism, and ageism for these individuals are biases that are not occurring in isolation from one another.^11–13^ For example, older Black women can be alternatively stereotyped as Black people, as women, as old, or as older Black women specifically. As follows, intersectionality is a comprehensive framework for understanding the compounding effects of being a part of more than one oppressed social group.^11–13^ One hypothesis (double-jeopardy) suggests that being a member of multiple negative social groups will have an additive affect, whereas a second hypothesis (Global Inhibition) suggests that whenever two biases exist, the more dominant bias will win out over the weaker biases.^12^ At present, the effect of these compounding intersecting biases on an aging population is unclear.

Younger adults, particularly millennials, tend to be the drivers of social change, which has led to an upending of traditional behavioral norms in the workplace.^14^ Millennials are projected to overtake baby boomers as the largest generation in history by 2028.^15^ Younger generations have opposed and confronted longstanding oppression and discrimination against individuals based on race and gender through various movements (*e.g*., Black Lives Matter, MeToo movement, The Women's March).^16^ This propensity to be on the forefront of evolving social norms will continue to increase diversity and societal heterogeneity in both public and private spaces.^16^ These younger individuals will ultimately determine the trajectory of existing biases. However, what is unknown is how the current younger population in the United States perceives older, racially, and ethnically diverse adults.

As the global population is aging and becoming more diverse at an accelerated rate, it is imperative to understand the attitudes and beliefs of younger adults toward diverse older populations.^17^ Therefore, it is paramount to identify biases in the current population and determine their potential influence on future health care disparities. We investigated the race and gender bias of individuals on older Black and White individuals using Amazon's Mechanical Turk (MTurk), a crowdsourcing marketplace that tends to include a predominantly millennial-aged population. In this study, we want to explore whether racial and gender identities of participants will have any significant influence on their perception of older Black and White men and women and whether the interaction of participant age group is associated with perceptions.

## Methods

### Photo database development

Owing to the limited age and racial/ethnic diversity of existing face databases such as the Chicago Face Database and AgeGuess, we developed a database of older, racially diverse photos for this study. This process has been described in a previous study for middle-aged models.^18^ We recruited adults 70+ years of age that identified as either Black or White race from a metropolitan city in Arizona to serve as models. Models were provided with matching apparel that consisted of gray t-shirts consistent with other face databases. We asked models to remove eyewear, jewelry, hair accessories, and hats. They were also instructed to maintain a neutral facial expression likened to a passport photo. We took 132 photos with a Nikon DX camera in portrait mode against a cloth white backdrop. Photos were then edited using Adobe Photoshop for consistent photo brightness and size. Unlike the Chicago Face Database, we did not adjust core facial features to make all facial features equal in size.^19^ Models provided written informed consent for their participation.

### Photo selection process

**FIG. 1.**
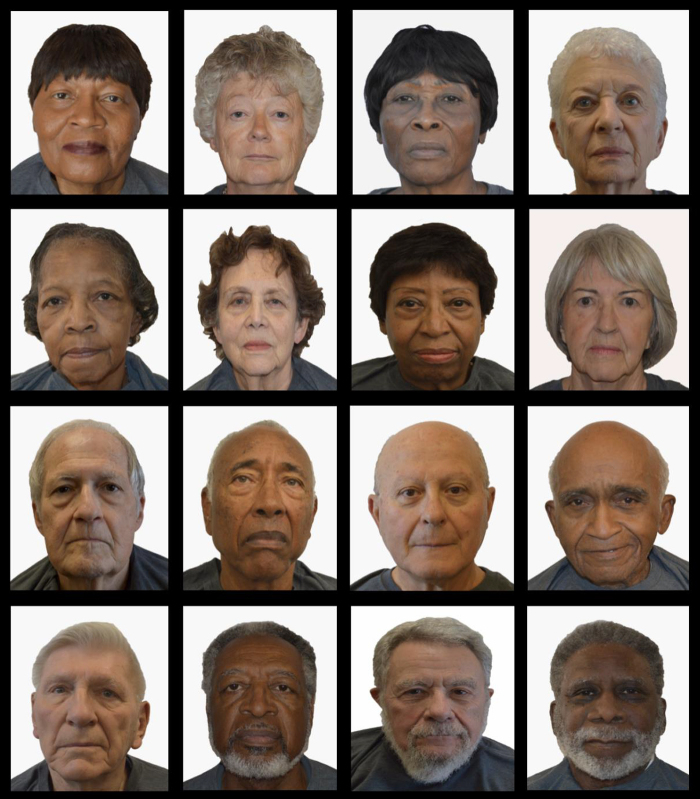
Photos of older adult subjects. These photos of subjects in their seventh decade were used in the MTurk survey. MTurk, Mechanical Turk.

The original set of 132 photos were categorized by individual study team members into subsets based on similarities in appearance: facial expression, hairstyle, facial features, and age group mid-70s. Over the course of several meetings, the study team (S.S., L.L., K.H.-T., L.Z., K.B.) narrowed the subset by consensus to 16 photos matching appearances of women to women (Black and White race) and men to men (Black and White race) (Fig. 1, right to left). This process helped isolate perceptions according to age, race, and gender and lessen confounders that may be present from variable expression, hairstyle, facial features, and age if all 132 photos were included. A duplicate of the final 16 photos was created with a caption stating that the patient has heart failure for a total of 32 photos. We did this as a case example of a person with disease because clinical comorbidities rise with age and may affect how older adults are perceived.^20^ Methods are similar to a prior study previously published.^18^

### Survey development

To investigate the implicit bias of participants based on physical attributes, we chose to focus only on older individuals. A survey was developed using Qualtrics survey software to identify biased perceptions of the models' photos. Questions about each photo included an estimation of the model patient's age, gender (male or female), race (Black, American Indian or Alaska Native, Asian, Native Hawaiian or Other Pacific Islander, or White), and characteristic using a Likert scale (1–10, strongly disagree to strongly agree) of the model as healthy, attractive, trustworthy, intelligent, and their facial expression (happy, neutral, sad, and threatening). At the conclusion of the survey, participants were asked basic demographic questions about themselves (age, gender, race, and ethnicity). Provisions were put into place to block duplicate responses and use of automated tasks software. The survey was piloted among both research professionals and laypersons to assess comprehension as described in a prior study using MTurk.^18^

### Survey participants

**FIG. 2.**
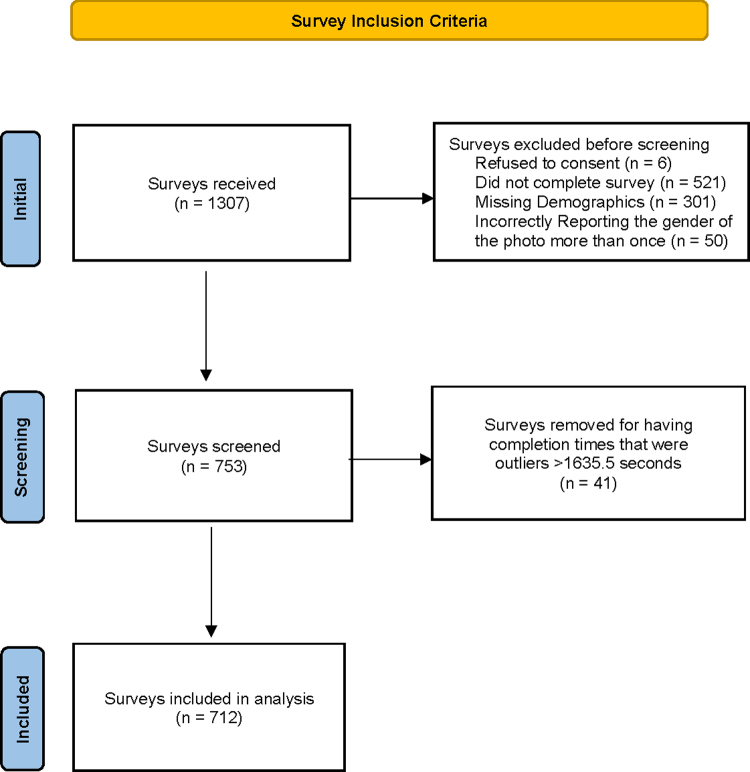
Survey inclusion criteria. Some surveys met more than one exclusion criteria. The total number of surveys excluded on all criteria was *n* = 595. When a participant reported the wrong gender for only one photo, the participant's entire survey was not removed; rather their responses were excluded for that individual photo. From the final sample of 712 participants, responses for a photo were excluded for 82 of them. This does not change the total number of surveys included in the analysis. Outliers for completion were defined by Tukey's Fences (less than quartile 1 − 1.5 [interquartile range] = −23.5 seconds, or greater than quartile 3 + 1.5 [interquartile range] = 1636.5 seconds).

Survey participants were recruited through Amazon's crowdsourcing marketplace MTurk from September 24, 2018 to September 26, 2018. MTurk workers are generally younger, college educated, and of White race.^21^ Participants 18 years of age or older and currently residing in the United States were eligible for the study. Informed consent was obtained electronically before any data collection occurred. Participants were randomized in Qualtrics to 16 of the 32 photos, which could include the same model with and without heart failure. Participants received $.07 for completing the survey. Participants were excluded for not completing the survey, refusal to consent, missing demographics, incorrectly reporting the gender of the photo more than once, and for having completion times that were outliers based on Tukey's Fences (Fig. 2). After applying these exclusion criteria, we arrived at our final set of 712 eligible participants, for whom the median time to complete the survey was 12.6 minutes. This study was reviewed and approved by the University of Arizona Institutional Review Board (Protocol No. 1804472883).

### Statistical analysis

Descriptive statistics (mean ± standard deviation [SD] or %) were conducted for participant demographics (*i.e*., age, gender, race, and ethnicity). In the unadjusted analyses, for each participant we first calculated the mean response for each characteristic (age, healthy, attractive, intelligent, trustworthy, neutral, happy, sad, and threatening) over Black photos, White photos, male photos, and female photos. Then for each participant we calculated the differences in mean rating of each characteristic between photos of men and photos of women and between photos of White individuals and photos of Black individuals. For example, for female participants, comparing response “sad” between photos of men and women we calculated the mean rating for “sad” for photos of men and the mean “sad” for photos of women, for each female participant.

We then calculated the difference in those means for each female participant. A *t*-test was used to test whether the mean of those differences was equal to zero or not. Next, for each participant the difference in the mean between races and between genders were calculated. Participant race and gender were delineated *via* participant self-identification. Owing to the randomization of photos presented, participants did not necessarily review equal numbers of photos for race, gender, and heart failure diagnosis. A Bonferroni correction was applied to *p*-values to adjust for 36 tests.

For the adjusted analyses, a mixed effects model was used to assess the effect of participant race/ethnicity, gender, and age-group and photo race and gender on each of the following characteristics: healthy, attractive, intelligent, trustworthy, neutral, happy, sad, and threatening. Each of the eight mixed models included fixed-effects for participant gender (Woman/Man), photo gender (Woman/Man), participant race/ethnicity (Non-Hispanic White/BIPOC), photo race/ethnicity (White/Black), participant age group (>37 and ≤37 years), centered on the mean, 37.81 years), an interaction of photo and participant gender, an interaction of photo and participant race/ethnicity, an interaction of photo race and participant age group, an interaction of photo gender and participant age group, and whether the person in the photo was said to have heart failure or not. The model also included random intercepts for participant and photo. The primary effects of interest are the two interaction effects (difference-in-differences). A Bonferroni multiple comparison adjustment for eight tests was applied to the confidence intervals and *p*-values for the interaction effects. All statistical analyses were performed using R Version 4.2.1,^22^ with statistical significance at an alpha level of 0.05.

## Results

### Participant demographics

Demographics for 712 eligible participants that completed the survey are presented in Table 1. The mean age of participants was 37.81 years (SD = 12.42). The majority of participants were women (70%), and the majority were non-Hispanic White (70%) race followed by BIPOC participants: Black (10%), Asian (8%), American Indian or Alaska Native (2%), and Native Hawaiian or Pacific Islander (<1%) race and ethnicity.

**Table 1.** Participant Demographics

	Female, *n* = 498 (69.9%)	Male, *n* = 214 (30.1%)
Age, mean (SD)	37.9 (12.4)	37.7 (12.4)
Non-Hispanic White	351 (70.5%)	144 (67.3%)
Non-Hispanic American Indian or Alaska Native	5 (1.0%)	6 (2.8%)
Non-Hispanic Asian	39 (7.8%)	16 (7.5%)
Non-Hispanic African American or Black	54 (10.8%)	16 (7.5%)
Non-Hispanic Native Hawaiian or Pacific Islander	2 (0.4%)	0 (0%)
Hispanic	47 (9.4%)	32 (15.0%)

SD, standard deviation.

### Unadjusted results by race

On average BIPOC participants rated photos of Black individuals as healthier and more attractive when compared with photos of White individuals (Table 2). On average White participants rated photos of Black individuals as sadder compared with photos of White individuals. Both groups rated photos of Black individuals as more trustworthy, happier, younger, less threatening, and less neutral compared with photos of White individuals. There were no statistically significant differences for rating of intelligence between photos of White and Black individuals for either group of participants.

**Table 2.** Black, Indigenous and People of Color and White Participant Ratings of Photos by Race and Female and Male Participant Ratings of Photos by Gender

All BIPOC participant ratings				Non-Hispanic White participant ratings		
	Black photos, mean ± SD	White photos, mean ± SD	*p*	Black photos, mean ± SD	White photos, mean ± SD	*p*
Age, years	73.56 ± 3.82	74.92 ± 4.30	<0.01	74.03 ± 3.75	74.81 ± 3.39	<0.01
Healthy	5.10 ± 1.45	4.83 ± 1.49	0.01	4.90 ± 1.34	4.83 ± 1.30	1.00
Attractive	5.19 ± 1.69	4.88 ± 1.74	<0.01	5.21 ± 1.77	5.09 ± 1.74	0.06
Intelligent	6.25 ± 1.30	6.13 ± 1.25	1.00	6.41 ± 1.31	6.36 ± 1.28	1.00
Trustworthy	6.26 ± 1.36	5.85 ± 1.38	<0.01	6.47 ± 1.35	6.18 ± 1.35	<0.01
Neutral	5.87 ± 1.38	6.40 ± 1.52	<0.01	5.90 ± 1.53	6.56 ± 1.60	<0.01
Happy	4.89 ± 1.36	4.20 ± 1.63	<0.01	4.63 ± 1.35	4.06 ± 1.63	<0.01
Sad	4.31 ± 1.54	4.33 ± 1.70	1.00	4.22 ± 1.51	4.01 ± 1.58	<0.01
Threatening	2.77 ± 1.72	3.37 ± 1.85	<0.01	2.52 ± 1.63	2.92 ± 1.74	<0.01

Women participant ratings				Men participant ratings		
	Men photos, mean ± SD	Women photos, mean ± SD	*p*	Men photos, mean ± SD	Women photos, mean ± SD	*p*
Age, years	74.51 ± 3.66	74.20 ± 3.62	0.65	74.53 ± 3.72	74.09 ± 3.58	1.00
Healthy	4.86 ± 1.37	4.81 ± 1.35	1.00	5.04 ± 1.37	5.05 ± 1.41	1.00
Attractive	5.08 ± 1.71	5.37 ± 1.71	<0.01	4.99 ± 1.71	4.82 ± 1.80	0.11
Intelligent	6.39 ± 1.26	6.36 ± 1.30	1.00	6.28 ± 1.18	6.16 ± 1.28	0.16
Trustworthy	6.23 ± 1.28	6.30 ± 1.36	0.78	6.23 ± 1.28	6.19 ± 1.34	1.00
Neutral	6.15 ± 1.46	6.37 ± 1.59	<0.01	6.01 ± 1.33	6.14 ± 1.40	1.00
Happy	4.47 ± 1.41	4.09 ± 1.53	<0.01	4.97 ± 1.39	4.43 ± 1.58	<0.01
Sad	4.02 ± 1.49	4.04 ± 1.59	1.00	4.40 ± 1.62	4.56 ± 1.59	0.16
Threatening	2.47 ± 1.50	2.69 ± 1.63	<0.01	3.30 ± 1.91	3.55 ± 1.90	<0.01

BIPOC, Black, Indigenous and People of Color.

### Interaction of participant race with photo race

**FIG. 3.**
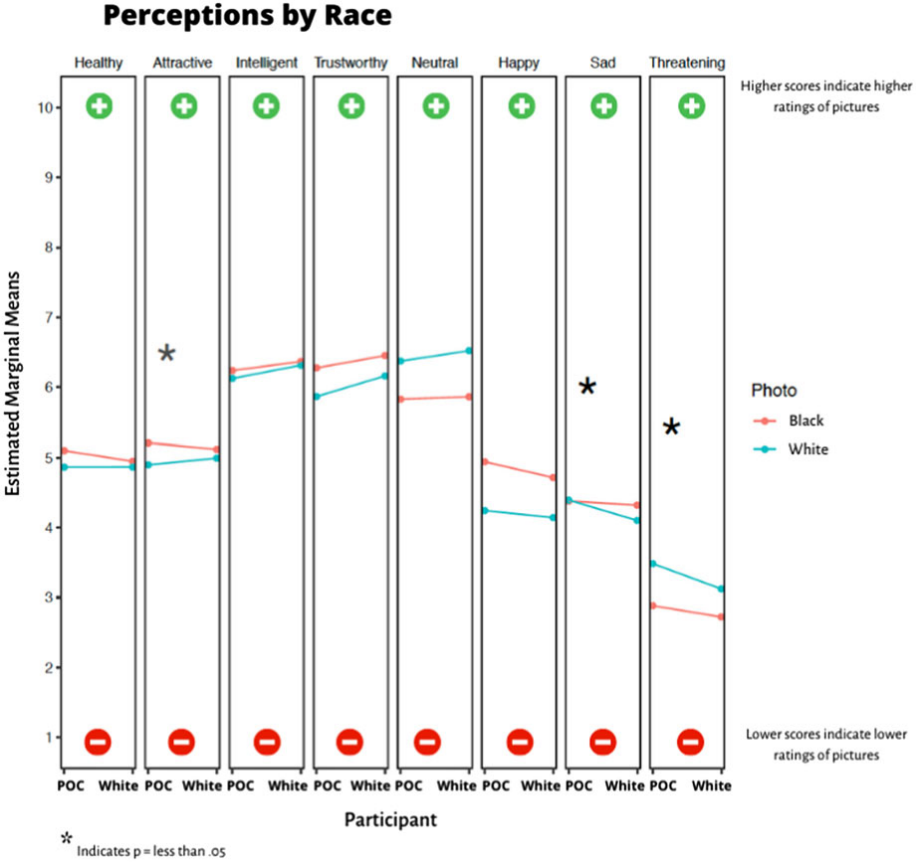
Interactions by race. Higher scores indicate higher ratings for photos, whereas lower scores indicate lower ratings for photos. *p-Value was <0.05 for attractive, sad, and threatening. BIPOC, Black, Indigenous and People of Color.

In the adjusted analyses, we observed that both BIPOC and non-Hispanic White participants on average rated photos of Black individuals as more attractive compared with photos of White individuals (Fig. 3). The difference in ratings of attractiveness between photos of Black individuals and photos of White individuals was significantly greater among BIPOC participants (race-by-race interaction *p* = 0.003). The difference in ratings of threatening between photos of Black individuals and photos of White individuals was significantly greater among BIPOC participants (interaction *p* = 0.009). Regarding sadness, BIPOC participants rated photos of Black and White individuals similarly, on average. White participants perceived photos of Black individuals as sadder compared with photos of White individuals. The difference in rating of sadness between photos of Black individuals and photos of White individuals was significant between BIPOC and White participants (interaction *p* = 0.016). Differences in rating between photos of Black individuals and photos of White individuals for perceptions of health, intelligence, trustworthiness, neutral, and happiness appearance did not vary significantly between BIPOC participants and White participants.

### Interaction of participant age group with photo race

**FIG. 4.**
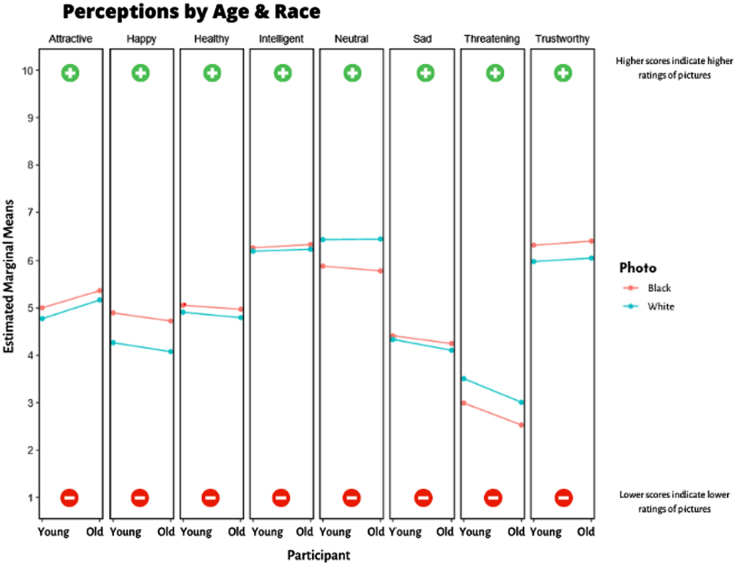
Interactions by age and race. Higher scores indicate higher ratings for photos, whereas lower scores indicate lower ratings for photos. No *p*-values reached statistical significance.

In the adjusted analyses, the average difference in rating between photos of Black individuals and photos of White individuals was similar for young and old participants for all characteristics (all *p*-values >0.05; Fig. 4).

### Unadjusted results by gender

Women rated photos of women as more attractive and neutral appearing compared with photos of men (Table 2). Both men and women perceived photos of men as happier and less threatening compared with photos of women. There were no statistically significant differences for other ratings between photos of women and men.

### Interaction of participant gender with photo gender

**FIG. 5.**
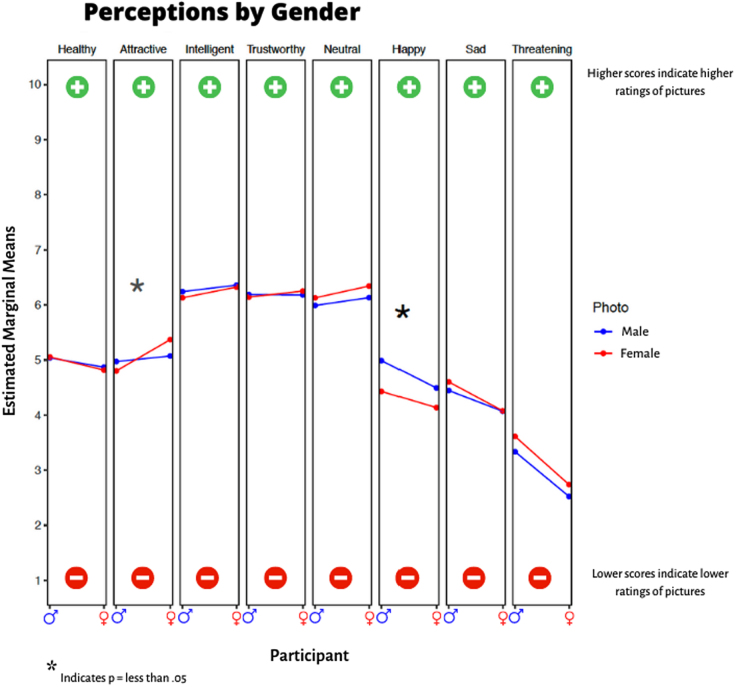
Interactions by gender. Higher scores indicate higher ratings for photos, whereas lower scores indicate lower ratings for photos. *p-Value was <0.05 for happy and attractive.

In the adjusted analyses, we observed a significant (*p* < 0.0001) gender-by-gender interaction in attractiveness with female participants rating photos of women as more attractive and male participants rating photos of men as more attractive (Fig. 5). Participants of both genders generally perceived men as happier, but the observed difference was larger among male participants (gender-by-gender interaction *p* = 0.026). No other statistically significant differences-in-differences were detected with photo and participant gender.

### Interaction of participant age group with photo gender

**FIG. 6.**
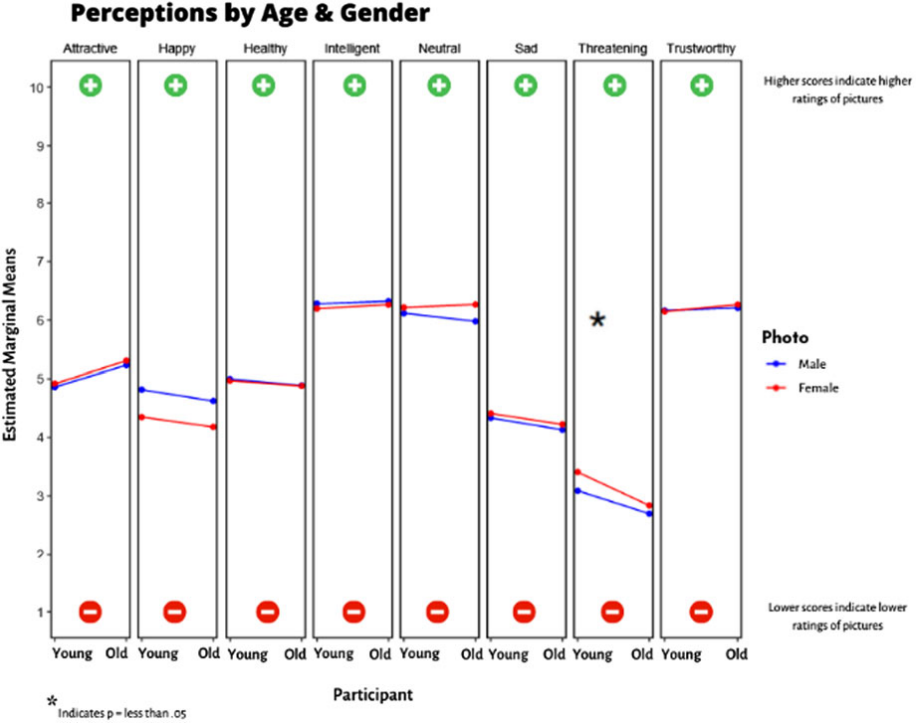
Interactions by age and gender. Higher scores indicate higher ratings for photos, whereas lower scores indicate lower ratings for photos. *p-Value was <0.05 for threatening.

In the adjusted analyses, we observed a significant (*p* = 0.012) age-by-gender interaction in threatening with both groups rating women as more threatening than men but with a larger difference among younger participants (Fig. 6).

## Discussion

This study consisted of a majority millennial-aged cohort with 56% of participants being 22–37 years of age at the time they took the survey. The rest of the participants' ages fell into the following groups: 27% were 38–53 years, 13% were 54–72 years, and 3% were younger than the age of 22 years. We observed that participants generally perceived Black individuals more favorably compared with White individuals, and that White participants perceived photos of Black individuals as sadder compared with White individuals. In fully adjusted analyses, both BIPOC and White participants perceived Black photos as more attractive and less threatening compared with photos of White individuals, whereas both younger and older individuals found women more threatening compared with men. In this study, participants' gender-based perceptions followed more traditional norms, favoring men.

Stereotypes of Black individuals have been portrayed in the media in a way that has systematically saturated the American zeitgeist as a form of structural racism.^23–26^ Therefore it would be reasonable to assert that some of the racial perceptions of Black individuals found in this study could be a result of these many years of manufactured public opinion. The perception in this study of older Black adults as nonthreatening and attractive could be a result of a stereotype that dates back decades.^24,25^ In an Oxford University Press journal article titled “Cinematic Racism: White Redemption and Black Stereotypes in … Films,” Hughey discussed the dehumanization of Black race to carnality and exoticism in young Black individuals and magical in older Black individuals.^24^

This media phenomenon combined with the increasing numbers of prominent older Black individuals in pop culture, may account for some number of individuals who perceive older Black individuals more favorably than older White individuals.^27^ Another potential explanation of these results is the global inhibition hypothesis, which has been observed in younger participants.^12^ This hypothesis posits that when an individual is a member of two competing stereotypes, the stronger stereotype will overrule the weaker stereotype.^12^ It could be the case here that the stronger stereotype, for this younger cohort, was that associated with older age rather than Black race. Another issue that was not examined in this study but could be present is colorism, where individuals with darker complexion are discriminated against more than those of lighter complexion.

The racial findings from this study are consistent with prior research that also observed favorable perceptions of older Black individuals from participants.^12,18^ A similar study by Solola et al found photos of older Black individuals in their sixties to be perceived as more trustworthy, more attractive, healthier, and less threatening compared with their White counterparts.^18^ One potential explanation of this cohort rating older Black individuals favorably could be the ever increasing diversity and open-mindedness of millennial-aged individuals. In a 2017 article, Milkman states “millennials have more progressive attitudes and beliefs than do older generations on a wide range of issues.”^16^ Perhaps the large millennial make up of this cohort, and the progressive views of millennial-aged individuals is resulting in favorable views of older Black individuals.

The stereotype that constructs individuals as being “magical” is typically an older Black individual, whereas younger Black individuals have been criminalized.^23,26^ These negative stereotypes, enforced by structural racism, date back to slavery and beyond, and may offer an explanation to the rating of sadness for older Black individuals by this cohort.^28^ In a journal article by Foster, the author explains how this stereotype demonizes Black individuals at intersections of race, gender, and class.^23^ White participants rating White individuals as less sad than Black individuals could also be explained by in-group bias. Automatic in-group bias is a preference for one's own group.^29^ Yet, widespread support of one's own race that is systematically empowered by U.S. society is also another form of supporting structural racism.

Both men and women participants perceived photos of their own gender as more attractive. Participants' bias toward their own gender may be owing to automatic in-group bias.^29^ This preference for one's own gender was stronger with women than with men, which is in alignment with previous research findings.^18,29^ Both men and women participants perceived photos of men as happier, and both young and old participants perceived women as more threatening. This suggests that gender bias does persist among this cohort of participants. Recent studies do affirm the presence of persistent gender biases, especially in stereotypically male dominated fields such as science, technology, engineering, and mathematics (STEM).^30,31^ What's more, biases also exist in fields where women are well represented.^31^ Begeny et al asked managers to evaluate an employee randomly assigned a male or female name.^31^ Both male and female managers evaluated male employees as more competent and recommended men receive a higher salary.^31^ Begeny et al found that the key drivers of these biases were those managers who thought bias was not happening.^31^ According to the authors, individuals who believe bias is not happening, tend to lack awareness in how discrimination may manifest that may inadvertently increase their susceptibility to expressing gender bias.^31^ This Begeny et al study illustrates that gender biases occur in various environments in both men and women alike. The intersectionality of being an older Black woman may be increasingly additive according to the Double-Jeopardy Hypothesis.^12^

Multiple studies have investigated the evolution of racial attitudes using survey tools.^3,4,18,29^ However, this study is one of the first, that we know of, to examine the racial and gender biases of millennial-aged individuals on older Black and White individuals using an online platform. Although other studies have found similar results, the application of the online platform provided access to a broader base of individuals throughout the United States.^18^ This study explores and expands the relationship between visual perceptions and bias. In an ever-expanding virtual world, racial discrimination is entering new realms and arenas including online virtual environments.^32^ In addition, bias is already built into artificial intelligence applications such as facial recognition software.^33–35^ With the rise of ethno-nationalism and the intersectionality of deep seeded gender bias, it is important to understand the biases of the millennial generation.^12,36–38^

Fortunately, millennials have demonstrated their propensity to drive social change, and oppose longstanding oppression and discrimination.^14,16^ This study's BIPOC cohort rated photos of older Black individuals as nonthreatening and attractive, whereas White participants rated photos of Black individuals as sadder. This result coupled with all participants finding photos of women to be less happy shows that we have more work to do in this arena.

These findings reinforce the need for policies that support women and BIPOC individuals as well as the significance of evidence-based anti-bias and anti-racist trainings in schools and workplace settings regardless of whether women and BIPOC individuals are well represented in those spaces or not.^39,40^ Future research should focus on whether these biases are worse in areas of the United States that resist any teaching of critical race theory in elementary schools and evidence-based anti-bias/anti-racist training in their institutions. Future research should also explore how we can use this knowledge of gender and racial perceptions to improve health care delivery for Black and female individuals in the United States.

### Limitations

The results of this study should be taken with a few considerations. Using the MTurk platform could have introduced potential threats to validity such as sampling bias given most of the participants self-identified as non-Hispanic White, are generally young, and likely more tech-savvy. Underrepresenting some racial and ethnic groups could have skewed the results toward the White participant category. The process of photo randomization could also impact the results. For example, if a large number of BIPOC participants received a disproportionate number of photos of White individuals with heart failure, that would impact the measured effect of “Health” in BIPOC participants.

It is also possible that participants' answers were influenced by awareness of the purpose of the study or current events surrounding racial and ethnic social injustices resulting in positively favorable ranking of Black photos. Another factor that may have influenced results are the various shades of gray in the photos owing to lighting at the time the photo was taken. Survey participants' environment including room lighting, screen resolution, and presence of additional people may have influenced a participant's response. However, it has been shown that data collected *via* social media or MTurk versus in-person are not significantly varied.^41^ Provisions were put into place to block duplicate responses and use of automated tasks software, but we cannot guarantee that those efforts were not undermined.

## Conclusions

This study investigates the perceptions of older adults older than the age of 70 years based on gender and race. A cohort of majority millennial-aged participants viewed photos of Black individuals more favorably than photos of White individuals. Despite a volatile racial landscape, this research shows racial attitudes and beliefs may be improving. Gender perceptions tended to follow more traditional gender stereotypes with all participants perceiving men to be happier and women to be more threatening. This may suggest that gender stereotypes may be more deeply rooted and more difficult to change. Investigation is needed to identify effective strategies to minimize the influence of racial and gender stereotypes in society.

## Abbreviations Used

BIPOC: Black, Indigenous and People of Color
MTurk: Mechanical Turk
SD: standard deviation
